# Medical Opinion and Movement

**Published:** 1906-06-02

**Authors:** 


### MEDICAL OPINION AND MOVEMENT.

The daily press made a good deal of stir about the posting of certain names outside the Edinburgh Royal Infirmary, excluding the practitioners in question from admission to its walls for a stated period. The matter caused some excitement on account of its novelty, but everything has been happily settled by the managers accepting an apology from the gentlemen immediately concerned and withdrawing the notices. It is not usually difficult to maintain adequate discipline in the residents' quarters of a hospital. Whilst offering our congratulations to those who have brought about this happy solution we may express a hope that nothing of the kind will ever occur again in connection with the Edinburgh school.

We are glad to notice that Mr. H. J. Tennant is endeavouring to secure a clause in Mr. Birrell's Education Bill constituting it the duty of every local education authority to make arrangements, in accordance with a scheme to be agreed by the Board of Education, for attending to the health and physical condition of all children educated in public elementary schools. If Mr. Tennant's clause be accepted the educational authorities in every community should become the guardians of the physical health and development of the majority of the children in this country. If we were to secure an average efficiency in this respect our population everywhere would cease to exhibit those evidences of degeneration which, in large towns especially, have made thoughtful observers fear that the English people may, as matters stand at present, speedily disappear.

The efforts now proceeding with a view to obtain a royal charter for the British Medical Association mark an epoch in its history, and should cause those responsible for its management to make it the occasion of a new departure in method. The success of the British Medical Association has in many respects been phenomenal, but one result of the new situation has been to bring to the front and to place in a position of influence some at any rate whose views are too narrow to warrant their holding any such a position. Up and down the country, and wherever a few thoughtful members of the profession meet, the usual topic in recent times has been the danger of converting this great organisation of the medical profession into something approaching that of a mere trades union. Petty restrictions upon individual effort, attempted interference with the freedom of action of its members, worrying and vexatious meddling with relatively trivial matters are things to be avoided. Otherwise the British Medical Association, despite its increasing numbers, must gradually fall into discredit and lose that position of influence, now claimed for it, which is due to the work and foresight of its originators. If it is one day to hold the position of director of medical opinion throughout the English-speaking world several recent proceedings must be ended and better methods substituted for them.

The world owes more probably to members of the medical profession than to any other profession known to man. Among the fruitful workers of the last half-century in medical matters Sir Constantine Holman must ever hold a first rank. During 50 years of honorary work in association with the British Medical Association, for which he last year received the gold medal for distinguished merit, the Surrey Medical and British Medical Benefit Societies, and Epsom College, the yield in good to others has been immense. For 19 years Sir Constantine has been the life and soul of Epsom College, during which period it has been greatly developed, until now it has attained so high a standard that it takes rank amongst the leading public schools of the country. Not only as an administrator, but as a financier, Sir Constantine has made his mark. The finances of Epsom College have been brought, thanks to him, from a position of weakness and anxiety to one of permanent strength, so that his life's work may entitle him to be considered as the founder of a new school for the medical profession, which will grow more and more important and useful as the years roll by. Fifty years of such services entitle Sir Constantine to seek rest in retirement, where he will carry with him the best wishes for his happiness and health of a multitude of people, including the whole of the members of the profession to which he has devoted his life.

One of the most satisfactory developments in medical education of recent years has been the steady progress made with the object of affording adequate instruction to post-graduates in this country. London led the way, and the courses of post-graduate study which are now open to practitioners in the metropolis have proved popular with the profession, and have done much to strengthen the ties between practitioners and consultants. We have never been able to understand how it is that the medical schools, which are often unable to afford adequate facilities for practical instruction to all their students, have failed to recognise their responsibility by opening their wards to all post-graduate members for after-study and clinical instruction. It is surely to the interests of the schools and their students, whether post-graduate or not, that every one of the latter should acquire an accurate practical knowledge of his profession before entering general practice. We have more than once urged the members attached to medical schools to agitate in favour of facilities for after-study, and we are glad to notice that the University of Edinburgh and the Royal Colleges there have co-operated in organising a scheme of post-graduate teaching at the Royal Infirmary and the Royal Infirmary for Sick Children during the last two weeks of September and the first week of October 1906. It is a gratifying fact that post-graduates attending these courses will be accommodated in the university halls. The more post-graduate instruction is extended in the United Kingdom the better, and the Edinburgh departure is welcome, and should lead to the adoption of a similar system at other universities and teaching schools.

